# Point-of-care thrombocyte function testing using multiple-electrode aggregometry in dengue patients: an explorative study

**DOI:** 10.1186/s12879-020-05248-4

**Published:** 2020-08-06

**Authors:** Wesley de Jong, Tri Pudy Asmarawati, Inge Verbeek, Musofa Rusli, Usman Hadi, Eric van Gorp, Marco Goeijenbier

**Affiliations:** 1 Department of Viroscience, Erasmus MC, Rotterdam, the Netherlands; 2 Department of Internal Medicine, Universitas Airlangga Hospital, Airlangga University, Surabaya, Indonesia; 3 Department of infectious diseases, Rumah Sakit Umum Daerah Dr Soetomo, Airlangga University, Surabaya, Indonesia; 4 Department of internal medicine, Erasmus MC, Rotterdam, the Netherlands

**Keywords:** Dengue, Multiplate, Haemostasis

## Abstract

**Background:**

Dengue virus (DENV) causes the hospitalisation of an estimated 500,000 people every year. Outbreaks can severely stress healthcare systems, especially in rural settings. It is difficult to discriminate patients who need to be hospitalized from those that do not. Earlier work identified thrombocyte count and subsequent function as a promising prognostic marker of DENV severity. Herein, we investigated the potential of quantitative thrombocyte function tests in those admitted in the very early phase of acute DENV infections, using Multiplate™ multiple-electrode aggregometry to explore its potential in triage.

**Methods:**

In this prospective cohort study all patients aged ≥13 admitted to Universitas Airlangga Hospital in Surabaya, Indonesia with a fever (≥38 °C) between 25 January and 1 August 2018 and with a clinical suspicion of DENV, were eligible for inclusion. Exclusion criteria were a thrombocyte count below 100 × 109/L and the use of any medication with a known anticoagulant effect, nonsteroidal anti-inflammatory drugs and acetyl salicylic acid. Clinical data was collected and blood was taken on admission, day 1 and day 7. Samples were tested for acute DENV, using Panbio NS1 ELISA. Platelet aggregation using ADP-, TRAP- and COL-test were presented as Area Under the aggregation Curve (AUC). Significance was tested between DENV+, probably DENV, fever of another origin, and healthy controls (HC).

**Results:**

A total of 59 patients (DENV+ *n* = 10, DENV probable *n* = 25, fever other origin *n* = 24) and 20 HC were included. We found a significantly lower thrombocyte aggregation in the DENV+ group, compared with both HCs and the fever of another origin group (*p* < .001). Low ADP AUC values on baseline correlated to a longer hospital stay in DENV+ and probable DENV cases.

**Conclusion:**

Thrombocyte aggregation induced by Adenosine diphosphate, Collagen and Thrombin receptor activating peptide-6 is impaired in human DENV cases, compared with healthy controls and other causes of fever. This explorative study provides insights to thrombocyte function in DENV patients and could potentially serve as a future marker in DENV disease.

## Introduction

Dengue virus (DENV), most likely the most important mosquito transmitted viral disease in the world, is endemic in South East Asia. The estimated number of clinical infections worldwide is 67–136 million [1], with an estimated 500,000 people requiring hospitalisation every year. For Indonesia, which has a population of over 260 million people [2], DENV accounts for up to 55% of febrile cases for which a visit to a healthcare professional is necessary and in which there is a potential need of hospitalisation [3]. Upon infection, patients can present symptoms ranging from a simple flu-like illness up to severe disease warranting hospitalisation due to shock and/or haemorrhage. No treatment, nor an effective vaccine is available for DENV, which means that those who develop a severe form of the disease rely on supportive care, mainly consisting of the maintenance of the body-fluid volume [4]. In densely populated areas of Indonesia, such as the Java Island, DENV outbreaks can severely stress the capacity of healthcare systems, due to the large number of persons simultaneously seeking medical care [5, 6]. Earlier studies tried to identify patients at risk of developing severe dengue by specific biomarkers, genomics, machine learning and early point-of-care ultrasound. However, many of these promising markers call for difficult, expensive or laborious techniques that are not practical in the current Indonesian healthcare setting [7–10]. For adequate DENV diagnosis, clinicians should rely on serological (IgM/IgG) and/or NS1 detection or molecular tools. However, these assays are not routinely available in outbreak areas or come with a certain delay. Currently, it is difficult to early differentiate uncomplicated dengue from those that will develop a severe form of the disease that warrants intensified in-hospital monitoring. Such approaches were previously studied in a specific combination of hematological parameters to differentiate between dengue and malaria in Thailand [11].

Excellent studies have explored the role of thrombocytes in viral infections [12–14]. Thrombocytes not only play a crucial role in primary haemostasis, they are also known to play an important role in inflammatory responses, host defense and vascular integrity [15, 16]. Thrombocytopenia is commonly observed in patient with a DENV infection. The magnitude of thrombocytopenia, or “drop” in platelets seems to strongly correlate with DENV severity. Thrombocytopenia in DENV is mainly the result of decreased platelet production in the marrow and increased platelet destruction [17]. Interestingly, bleeding also occurs in DENV patients with thrombocyte counts within normal range, suggesting there is an important role for alterations in thrombocyte function or activation [18]. For thrombocyte activation, this leads to deposition of aggregates in microvascular structures. In this context, a number of surface markers such as P-selectin and CD63 expression, have been studied and these correlate to a decline in thrombocyte counts. Thrombocyte activation was found to be at a maximum after DENV clearance, indicating the presence of continuing thrombocyte activation mechanisms in the convalescent phase of the disease [12, 15].

Clinical studies of hospitalised DENV patients showed correlations between the occurrence of bleeding and thrombocytopenia and the need for a prolonged hospital stay [19]. During this early clinical phase, impaired thrombocyte function is not detected in routinely tests, but altered aggregation might relate to duration of hospital stay. To test this hypothesis, bench-top tests to assess thrombocyte function that are characterized by a short turn-around time would be suitable. In this matter, point-of-care thrombocyte aggregation tests are extensively used in the field of cardiology and cardiothoracic surgery [20], while a small number of studies are looking at platelet function in neurology [21], gynaecology [22] and sepsis [23]. Furthermore, patients presenting with Haemorrhagic Fever and Renal Syndrome (HFRS) due to Puumala orthohantavirus infection, showed impaired thrombocyte aggregation on almost all test reagents when bedside platelet aggregation was assessed using Multiplate® [24]. The Multiplate® analysis is based on the principle that platelets become sticky upon activation by reagents and adhere. These aggregate onto metal sensor wires in the Multiplate® analyser, which then measures an increase in electrical resistance. Over a six-minute timeframe, aggregation units (AU) are plotted against time, resulting in an Area Under the aggregation Curve (AUC), in Units. Adenosine diphosphate (ADP) is an important general agonist for platelet aggregation [25]. Collagen mediates the integrity of the vascular wall and its actions prevent excessive hemorrhage or thrombosis [26]. Last, thrombin receptor activator for peptide 6 (TRAP-6) acts via thrombin receptor protease-activating receptor-1, which is highly expressed on platelets [27].

Clinicians in low- to middle income countries where DENV is endemic, usually rely on the WHO 2009 case description to estimate the clinical course of a patient [28, 29]. If locally available, additional rapid DENV non-structural protein 1 (NS1) tests play a limited role in the decision-making process, especially taking into account that some patients present quite late to the hospital, when the virus was already neutralised and hence no NS1 is present. ELISA-based testing is known to have better characteristics, but it is more laborious and is only suitable for batch testing. As stated previously, additional understanding of thrombocyte function in DENV infections would be of value for healthcare settings in Indonesia, or indeed elsewhere.

In this study we explore the use Multiplate™ multiple-electrode aggregometry to evaluate its potential as marker of DENV severity expressed by the duration of hospital stay in the very early phase of DENV infectionin Indonesia.

## Methods

### Patients and controls

Consecutive patients were included in the study, which ran from 25 January to 1 Augustus 2018 at the department of internal medicine of the Universitas Airlangga hospital in Surabaya, East-Java, Indonesia. Patients aged 13 or older, presenting with a fever (≥ 38 °C) on the ward or Emergency Department, and for whom there was a clinical suspicion of DENV infection in accordance with the case definitions in the WHO Dengue 2009 guideline [28], were deemed eligible for inclusion. Exclusion criteria were a thrombocyte count below 100 × 10^9^/L and the use of any medication with a anticoagulant and/or antiplatelet effect, including nonsteroidal anti-inflammatory drugs (NSAIDs), acetyl salicylic acid (ASA), non-vitamin K oral anticoagulants (NOAC), direct oral anticoagulants (DOAC) and antiplatelet drugs such as clopidogrel, dipyridamole or vitamin K antagonists. Also, patients with inherited platelet function disorders (e.g. Von Willebrand disease) were excluded. After obtaining written informed consent from patients or their legally authorised representatives, we collected blood samples on baseline, day 1 and 7 (+/− 48 h). Patients were followed up according to local practices and guidelines. Based on the clinician’s discretion, this included imaging, urinalysis and blood culture.

### Sample collection

All samples were drawn using a BD vacutainer system using 0.7 mm × 25 mm needles, and tubes were gently inverted five times after blood was drawn. Multiplate samples were drawn to Diapharma Multiplate® Hirudin Blood Tubes. At inclusion, an additional BD Vacutainer® SST™ serum tube was taken and spun down in a centrifuge at 2000 RCF (room temperature) for 15 min.

### Dengue virus diagnostics and study group assignment

Panbio® Dengue Early Rapid test was used to identify dengue cases by ascertaining the presence of the NS1-antigen in the serum. This information was used to track the number of patients in each group throughout the study. Residual serum was aliquoted, and stored at minus 80 °C while awaiting further processing. Stored serum samples were tested at a later stage with a Panbio® Dengue Early ELISA dengue NS1 antigen capture ELISA, according to the manufacturer’s specifications. In case of a positive NS1 ELISA, patients were assigned to the study group DENV-confirmed. Consequently, patients were assigned to “fever of another origin” in case of a clear working diagnose other than DENV. Others were evaluated against WHO 2009 guidelines [28] and classified in the DENV probable group. Full criteria are available from Appendix A. For logistical reasons, no convalescent samples could be collected for IgM/IgG testing.

### Platelet aggregation measurement using multiplate

Using Multiplate, Hirudin blood tubes were tested using ADP and TRAP-6 reagents, as per the manufacturer’s instructions and COL reagent. In brief: ADP and TRAP-6 reagents were reconstituted with 1000 μL high purity distilled water and aliquoted in vials of 110 μL. These reagents were stored at minus 80 °C for a maximum of 4 weeks, then while they were in use kept at between 2 and 8 °C for a maximum of 7 days. COL reagent (CHRONO-LOG® corporation, P/N 385) was prepared by mixing 50 μL COL with 450 μL isotonic glucose solution (pH 2.7–2.9). The prepared COL reagent was used for a maximum of 1 week and kept at between 2 and 8 °C. Using 20 μl of the solution in a Multiplate test cell, the final concentration achieved was 3.2 μg/ml. Blood testing in the Multiplate was done a maximum of 4 h after blood draw. To start the Multiplate measurements, 300 μL of sodium chloride 0.9% solution was mixed with 300 μL of Hirudinized whole blood at 37 °C for 3 min, after which 20 μL of the reagent (either ADP, TRAP or COL) was added. For the statistical analysis, tests were performed in duplo, using the mean of two similar tests (i.e. same patient/reagent). Due to logistical reasons, not all samples that were available could be tested with COL reagent (see the results).

### Statistical analysis

Based on earlier work by Peerschke and colleagues, to detect a hypothesised AUC 20 difference in baseline means between study groups with a power of 80%, we calculated the following sample sizes: ADP *n* = 27, TRAP *n* = 20 and COL *n* = 30 respectively [30]. Statistical analysis was performed using IBM SPSS statistics software (for Windows, version 25). Normality testing was performed using Shapiro-Wilk (with a value of .05 or above considered as normally distributed data). If the data was distributed normally, the means were compared with a t-test, or else using a Kruskal-Wallis and Mann-Whitney U-test. Ethical clearance was obtained from the ethical committee of the Universitas Airlangga hospital, with reference 126/KEH/2017, and all study procedures were conducted following the Declaration of Helsinki [31].

## Results

### Study-group assignment

**Fig. 1:**
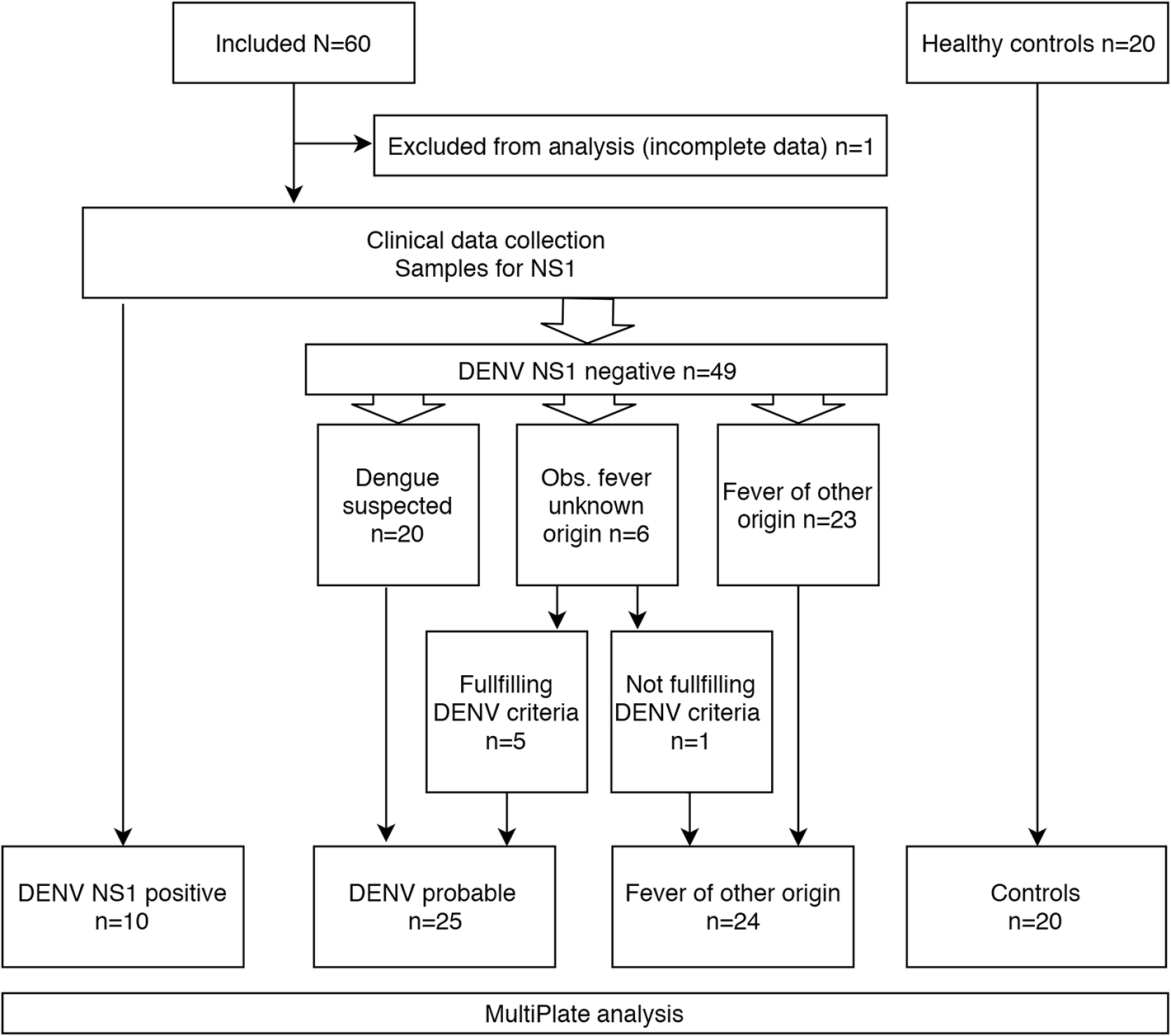
Flowchart for the analysis of baseline (day of inclusion) samples following the CONSORT 2010 Statement^1^. The data shown for DENV NS1 positive is based on the DENV NS1 ELISA results (see methods, Appendix A and Supplementary Table 1.). DENV criteria following WHO 2009 Dengue guidelines. 1| Schulz KF, Altman DG, Moher D, for the CONSORT Group. CONSORT 2010 Statement: updated guidelines for reporting parallel group randomised trials. BMC Medicine 2010, 8:18

We enrolled 60 subjects; all of them were laboratory tested for the presence of DENV NS1 antigen in serum. Figure 1. shows an overview of the enrolment of the participating patients, who were subsequently assigned to the different groups. During the study period, 9 patients initially tested positive using the DENV NS1 rapid test, while 1 additional DENV case was later confirmed by using the more sensitive DENV NS1 ELISA (totalling 17% of all the subjects, see Supplementary Table 1.). We assigned 59 patients to the following study groups: DENV-confirmed (*n* = 10), DENV-probable (*n* = 25) and fever of another origin (*n* = 24). For reasons of incomplete data, 1 patient was excluded from the analysis. A group of healthy hospital workers served as the control population (*n* = 20).

### Baseline study and disease characteristics

**Fig. 2:**
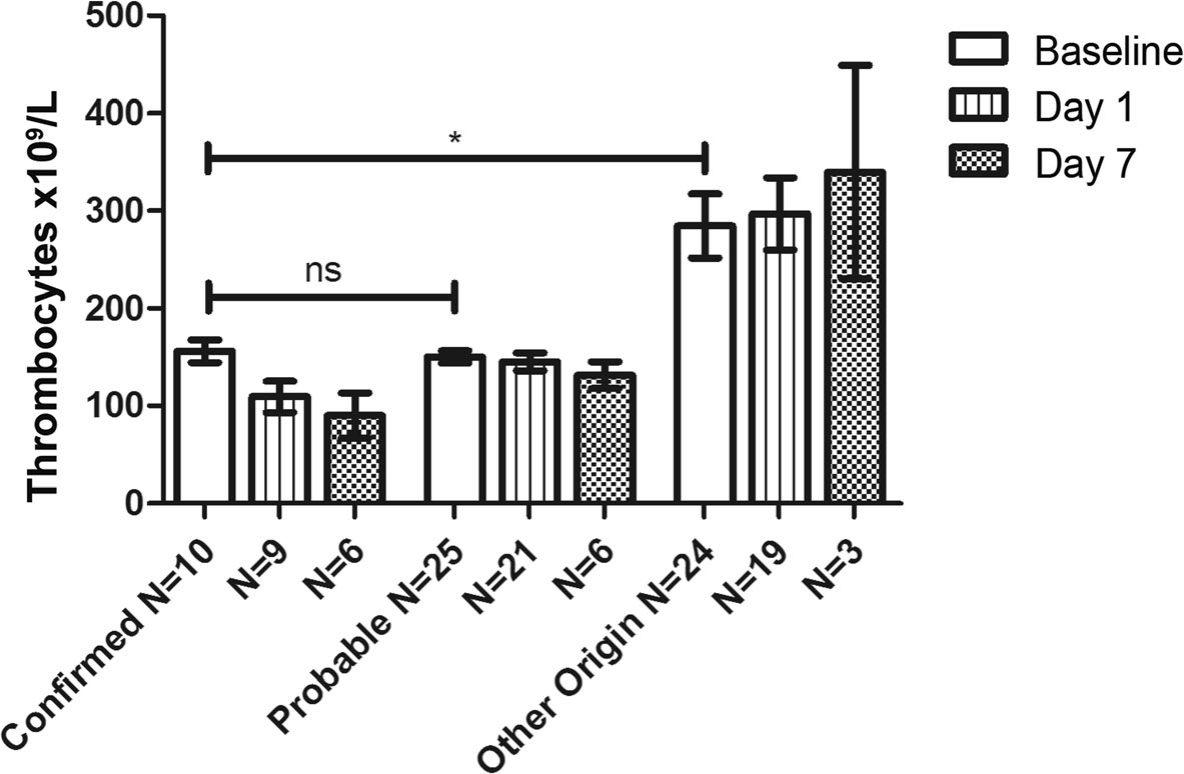
Thrombocyte counts (× 10^9^/L) presented as Mean/Standard Error of the Mean (SEM) for baseline (day of inclusion), day 1, day 7 (+/− 48 h or at discharge). *N* = represents number of samples available. Data for Confirmed and Other Origin groups is not normally distributed. No data available for healthy controls. Statistical analysis using Mann-Whitney U-test. * = significant *p* = .0173; ns = not significant

**Fig. 3:**
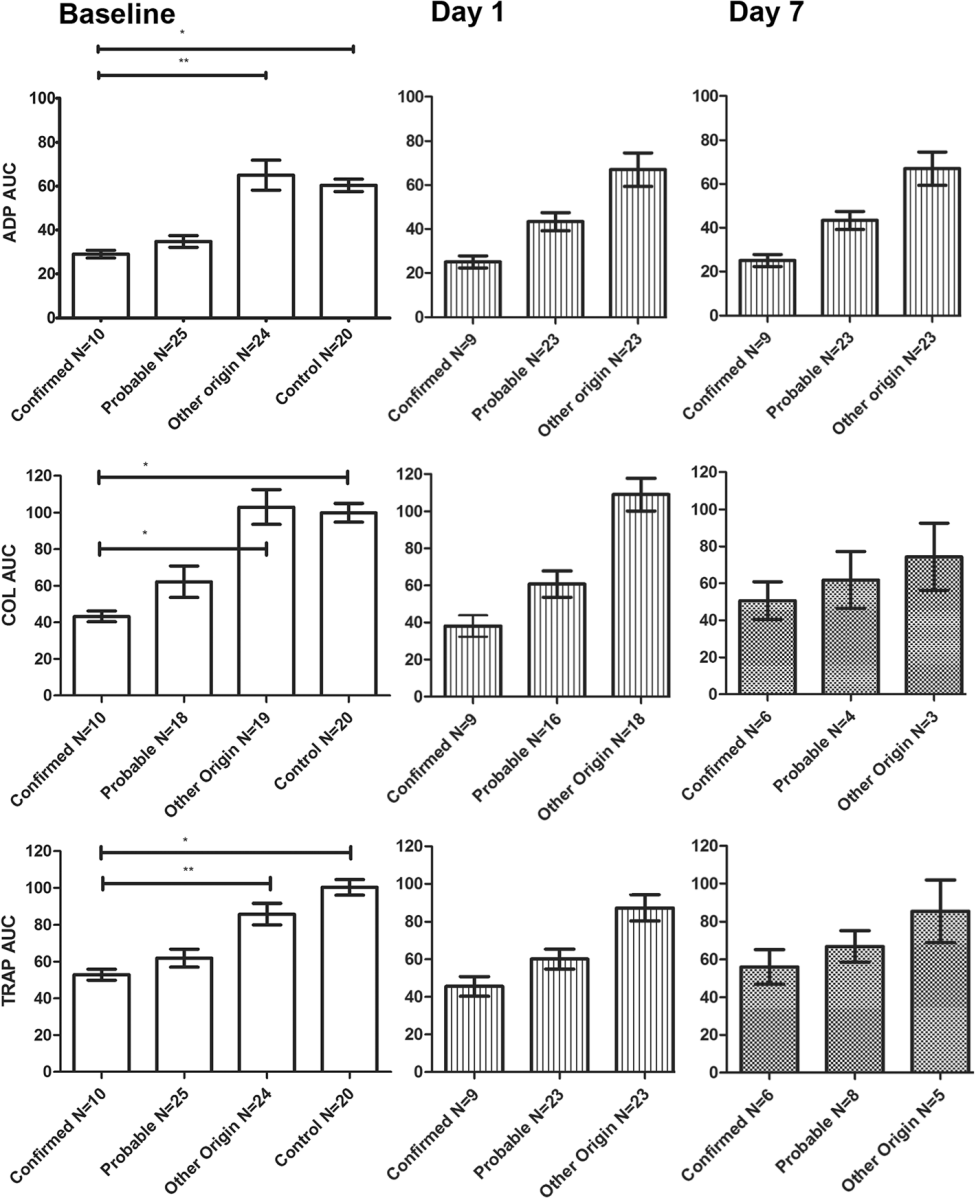
Area Under the Curve (AUC) for stimulation of the thrombocyte adenosine diphosphate (ADP) receptor, collagen (COL) induced aggregation of thrombocytes and stimulation of thrombin receptor activating peptide-6 (TRAP-6). Data shown as mean with standard error of the mean (SEM). The number of samples analysed/available is shown under the x-axis . and were influenced by discharge of patients (clinicians’ discretion). * *p* < .0001; ** *p* = .0005. Statistical analysis using Mann Whitney U-test

Baseline and clinical characteristics are shown in Table 1. With a mean age of 19, the DENV-confirmed group was significantly younger than that of the fever of another origin group (mean age 28), U = 34, *p* = .001). The mean, self-reported fever duration was 3.60 days for DENV-confirmed cases, 4.64 for DENV-probable (U = 70, *p* = .039) and there was a mean duration of 6.63 days for those with a fever of another origin (U = 67, *p* = .043). Subjects classified as having a fever of another origin presented with complaints suspected for: a respiratory-tract infection (*n* = 5, 20%), typhoid fever (*n* = 9, 38%), a urinary-tract infection (*n* = 2, 8%), other abdominal focus (*n* = 4, 17%) and undifferentiated fever/sepsis (n = 4, 17%). We observed the presence of DENV signs and symptoms in all study groups. Leukocyte counts for DENV-confirmed cases were significantly lower compared with both DENV-probable (a mean of 3.24 × 10^9^/L vs 5.56 × 10^9^/L (U = 46 *p* = .004)), as well as a fever of another origin (a mean of 10.72 × 10^9^/L U = 11 *p* < .001). As shown in Fig. 2., the mean thrombocyte count for DENV-confirmed cases was 156 × 10^9^/L, while for DENV-probable cases it was 150 × 10^9^/L and for a fever of another origin it was 284 × 10^9^/L (U = 56, *p* = .016 for DENV-confirmed vs a fever of another origin). No subjects showed signs of shock on enrolment, as the mean arterial pressure (MAP) was ≥65 mmHg for all. The mean duration of hospital stay, following the clinicians discretion, for all subjects was 2.05 days, with a range of 0 to 8 days and no significant differences between groups (DENV-confirmed vs DENV-probable *p* = .051 U = 74, DENV-confirmed vs Other origin *p* = 0.152 U = 83). While not significant, it should be noted that the mean hospital stay duration for the DENV-confirmed group, i.e. those that tested NS1 positive on baseline, was 2.5 days, as opposed to 1.8 for the DENV-probable group – which is reflected by a decline in numbers analysed as shown in Fig. 3. One subject died (cause unknown, from the fever of another origin group) and one subject was referred for third-line treatment in another hospital (reason unknown).

**Table 1:** Baseline study characteristics

	Study population ***N*** = 59	Dengue NS1 confirmed ***n*** = 10	Dengue probable ***n*** = 25	Fever of another origin ***n*** = 24	Control group ***N*** = 20	***p***-value
Mean age (range)	28 (14–71)	19 (14–36)	24 (14–54)	35 (14–71)	30 (21–46)	<.05*
Male sex *n* (%)	30 (50.8%)	3 (30%)	16 (64%)	11 (45.8%)	9 (45%)	.271
Duration of fever days (range)	5.27 (1–30)	3.6 (2–4)	4.6	6.63 (1–30)	N/D	.058
Headache *n* (%)	39 (66.1%)	9 (90%)	19 (75%)	11 (45.8%)	N/D	.018°
Retro-orbital pain	4 (6.8%)	0 (0%)	4 (16%)	0 (0%)	N/D	.054
Nausea, vomiting	50 (84.7%)	8 (80%)	22 (88%)	20 (83%)	N/D	.812
Rash	3 (5.1%)	1 (10%)	1 (4%)	1 (4.2%)	N/D	.740
Swollen glands	2 (3.3%)	1 (10%)	0 (0%)	1 (4.2%)	N/D	.324
Aches and pains	18 (30.5%)	2 (20%)	9 (36%)	7 (29.2%)	N/D	.639
Mean MAP° (range)	90 (67–125)	89 (76–107)	97 (67–121)	92 (69–125)	N/D	.689
Mean temperature (range)	38.3 (36.0–40.0)	38.3 (36.8–39.4)	38.4 (36.6–39.7)	38.3 (36.0–40.0)	N/D	.962
Mean leukocyte count (range)	7.27 (1.24–22.94)	3.24 (1.24–6.17)	5.56 (1.88–8.90)	10.7 (3.72–22.94)	N/D	<.05*
Mean thrombocyte count (range)	205 (101–815)	156 (101–196)	150 (111–210)	284 (123–815)	N/D	<.05*
Leukocytosis	13 (22.0%)	0 (0%)	0 (0%)	13 (54.2%)	N/D	<.05*
Leucopenia	13 (22.0%)	8 (80%)	5 (20%)	0 (0%)	N/D	<.05*
Thrombocytosis	4 (6.8%)	0 (0%)	0 (0%)	4 (16.7%)	N/D	.044*
Thrombopenia	24 (40.7%)	4 (40%)	14 (56%)	6 (25.0%)	N/A	.087
Increased creatinine^+^ n/N (%)	2/38 (5.2%)	0/6 (0%)	2/21 (9.5%)	0/11 (0%)	N/D	N/C
X-ray suspected pneumonia n/N (%)	3/15	N/D	0/3 (0%)	3/12 (25%)	N/D	N/C
Urine dipstick/culture positive n/N (%)	7/11 (63%)	1/1 (100%)	0/4 (0%)	6/6 (100%)	N/D	N/C
Blood culture positive n/N (%)	1/1	N/D	N/D	1/1 (100%)	N/D	N/C
Duration of hospital stay in days (range)	2.05 (0–8)	2.50 (1–4)	1.80 (1–4)	2.13 (0–8)	N/D	N/C

Patients with fever on admission were scored for anamnestic parameters according to the WHO dengue 2009 guideline. Cases that were not NS1-confirmed were considered probable dengue on condition that at least two of the following symptoms were presented: headache, retro-orbital pain, muscular/joint pain, nausea/vomiting, swollen glands, rash, leukopenia. Continuous and semi-continuous data was analysed using the Kruskal-Wallis test and categorical data was analysed using the Chi-Squared test. Leukopenia defined as leukocyte count < 3.6 × 10^9^/L; Leukocytosis defined as leukocyte count > 11.0 × 10^9^/L; Thrombocytopenia defined as thrombocyte count < 150 × 10^9^/L; Thrombocytosis defined as thrombocyte count > 400 × 10^9^/L N/D not done; N/C not calculated (for limited data); In case of incomplete data, data is shown as number/Number evaluated (n/N); * significant (*p* < .05); ° Mean Arterial Pressure; ^+^incomplete data for 21 cases

### MultiPlate analysis

As shown in Fig. 3, MultiPlate analysis was available for ADP, COL and TRAP reagents in 59 (100%), 47 (80%), and 59 (100%) of the subjects, respectively, at baseline. The Area Under the aggregation Curve (AUC, in Units or U) for ADP, TRAP and COL on baseline was significantly lower for both DENV-confirmed vs Healthy controls (*p* < .001 for all analyses) and for DENV-confirmed vs fever of another origin (p < .001 for all analyses).

Meanwhile, Table 2. shows the relationship between baseline ADP, TRAP and COL measurements and the final duration of hospital stay. Firstly, using Spearman’s rank-order correlation, we found no correlation for ADP, TRAP, COL measurements at baseline for DENV-confirmed versus length of stay (ADP: rs(10) = − .065., *p* = .858; TRAP: rs(10) = −,.241, *p* = .503; COL: rs(10) = .085, *p* = .816) or for DENV-probable versus length of stay (ADP: r_s_(25) = − .371., *p* = .068; TRAP: r_s_(25) = − .299, *p* = .147; COL r_s_(18) = − .296, *p* = .232). Secondly, however, given that it was considered highly likely that subjects in the DENV-probable group indeed had a DENV infection and were clinically followed-up as such, we merged both DENV-confirmed and DENV-probable groups. We found that a lower ADP AUC correlated to a hospital stay of > 1 day (ADP: r_s_(35) = − .360, *p* = .033). For TRAP and COL, this effect was not observed (TRAP: r_s_(35) =−,321, *p* = .060; COL r_s_(28) = − .305, *p* = .115).

**Table 2:** Predictive value of low ADP on admission for hospital stay > 1 day

	Hospital stay < = 1 day or above		***p***-value	Hospital stay < =2 days or above		***p***-value
	Stay <= 1 day	Stay > 1 day		Stay <= 2 days	Stay > 2 days	
ADP n	12	23		26	9	
ADP mean (IQR)	40.25 (29.25)	29.39 (10.50)	.008**	35.54 (15.63)	26.11 (10.00)	.056*
TRAP n	12	23		26	9	
TRAP mean (IQR)	66.54 (37.38)	55.63 (23.00)	.123*	62.84 (25.00)	49.33 (14.75)	.055**
COL n	10	18		20	8	
COL mean (IQR)	65.47 (37.00)	49.94 (19.50)	.099*	59.66 (32.25)	45.06 (14.25)	.500*

Comparison of mean baseline (T1) Area Under the Curve values for ADP, TRAP and COL for patients with either confirmed dengue or probable dengue (grouped together, n = 35) to the length of hospital stay. * P-values calculated using Mann-Whitney U test. **Normally distributed data (Shapiro-Wilk > .05), statistical analysis using independent samples t-test

### Validity of MultiPlate results versus thrombocyte counts

The internal validity of results reported in the MultiPlate analysis depends on the reported thrombocyte counts (see discussion). Moreover, as mentioned earlier, compared with the fever of another origin group, thrombocyte counts were significantly lower in the DENV-confirmed group. Furthermore, the same holds true for ADP, TRAP and COL AUC. Using Spearman’s correlation we checked whether thrombocyte counts at baseline correlated to ADP AUC at baseline. While we found no correlation for DENV-confirmed and DENV-probable rs(35) = .231, *p* = .181, we did find a significant positive correlation for the fever of another origin, rs(24) = .765, *p* < .001.

### Clinical implementation

With the data reported in the previous paragraphs, we could potentially provide additional decision-making rules based on information that is readily available to the treating physician. First of all, a binary linear regression analysis was run to calculate odds-ratios for assignment to the DENV-positive/DENV-probable group, based on ADP values on baseline, the presence of leukocytopenia (< 3.6 × 10^9^/L) and the presence of thrombocytopenia (< 150 × 10^9^/L). For this analysis, cut-off values for ADP AUC baseline of < 40, < 35 and < 30 were chosen. As can be deducted from Fig. 3., as well as the calculations mentioned earlier, in the current study this range of ADP AUC values would seem to be specific to DENV patients. DENV-confirmed/DENV-probable could be predicted from the whole study population (72.9% correct) by using an ADP cut-off of < 40 (β = − 2.189 *p* = .003) and establishing the presence of leukocytopenia (β = 20.410 *p* = .998) and thrombocytopenia (β = .384 *p* = .611). If there is no leukocytosis (i.e. leukocyte count < 11.0 × 10^9^/L), the overall percentage of correct study-group prediction in the model increases to 83.1%, with the following characteristics: ADP cut-off of < 40 (β = − 2.383 p = .003), leukocyte count < 11 × 10^9^/L (β = 21.878 p = .998), thrombocytopenia (and leukocytopenia or normal leukocyte range (β = 22.18, p = .998), constant (β = − 17.206 *p* = .999). Secondly, to further simplify the model, we found that presenting with a headache as a clinical parameter with ADP AUC values could predict 79.7% of the cases (ADP < 40 β = − 2.669 *p* < 0.001, headache β = 1.459 *p* < 0.043, constant β = 3.429 *p* < 0.003).

### Trends in MultiPlate values on follow-up

Additionally, Fig. 3. also shows ADP (3a), COL (3b) and TRAP (3c) values during follow-up at day 1 and day 7 (+/− 48 h). Due to the discharge of subjects, the number of available follow-up samples is limited. In the fever of another origin group, AUC values for ADP, COL and TRAP are stable (not significantly different) during follow-up. For the DENV-probable group, a significant recovery of ADP is observed from baseline to day 1 follow-up (a mean ADP AUC of 34.85 at baseline and 43.37 at day 1 follow-up respectively; *p* = .009 using paired samples t-test). For COL and TRAP this was not the case (tested with paired samples t-test for all time points and study groups).

## Discussion

We assessed thrombocyte aggregation in acute DENV cases using multiple-electrode aggregometry (MultiPlate). Our data suggests that UAC values of ADP are not only significantly lower in acute DENV cases, but that they might also be associated with a prolonged hospital stay and could therefore support the estimation of disease severity. To the best of our knowledge this is the first study that has applied short-turnaround time thrombocyte aggregation tests in acute DENV cases. This explorative data contributes to the understanding of thrombopathies as result of a DENV infection and we suggest that thrombocyte aggregation tests, such as the MultiPlate, could be further studied in the clinical assessment of DENV-suspected patients.

The presence of DENV non-structural protein 1 (NS1) antigen in blood is known to relate to the early stages of DENV [32]. Subjects classified as “DENV-probable” in the current study, comply with the WHO DENV case description [28], but tested negative for NS1. This could be explained by the potential neutralisation of the NS1 antigen in re-infected patients in a highly endemic area, the diagnostic inaccuracy of the available tests, or a delayed clinical presentation – as subjects classified as “DENV-probable” had a longer self-reported duration of fever, compared to the “DENV-confirmed” subjects. Diagnostic methods with high sensitivity and specificity for the detection of DENV NS1, including reverse transcriptase PCR (RT-PCR), were not available here [33] and no convalescent samples with sufficient time-intervals (i.e. to detect fold rise in antibody titers) were collected for logistical reasons. The alternatively used enzyme-linked immunosorbent assay (ELISA) for DENV NS1 detection [34] marginally outperformed the NS1 rapid testing in our study. In future, point-of-care approaches with a lower limited of detection, might find its way into resource limited settings, and could be used instead [35]. Circulating DENV serotypes that the authors were not aware of during the study period might have affected the accuracy and overall result found, because, as was previously shown, compared with other serotypes the sensitivity of detecting NS1 for DENV-2 and DENV-4 serotypes, in particular, is somewhat limited [36]. This might explain why the number of confirmed DENV cases doesn’t correspond with other studies. These report a higher number of confirmed DENV (albeit, using more extensive diagnostic assays) in similar settings of up to 72% [37, 38]. Furthermore, according to recent data from Surabaya, in studies conducted by Wardhani et al., up to 66% of adults admitted with fever tested positive to the DENV NS1 rapid test [39]. Based on a recent multicenter observational cohort study, the number of severe dengue cases in Indonesia is as low as 2.3% [40]. The small number of participants in our study thus limits the occurrences of severe dengue cases. In addition, possible selection bias might have impacted both the numbers shown in these studies and the numbers that we report, because the design of the study meant that we had to rely on less sensitive, point-of-care testing.

In several studies, the complex role played by NS1 in cytokine release, endothelial disturbances and complement activation is discussed, as well as the both protective and detrimental effects attributed to NS1-specific antibodies during a secondary disease episode [41]. As this was not part of our objectives and given the small numbers in our study, no conclusions have been drawn from NS1 positivity and its relation to disease stage (i.e. the tendency, or otherwise, of developing bleeding complications) versus MultiPlate results. Clearly, the “fever of another origin” group is both anamnestic as well as biologically different, as is characterised, for instance, by a thrombocytosis and leukocytosis. As the authors are not acquainted with any other research on viral cases and usage of MultiPlate, besides the studies of Laine and colleagues [24] on Puumala orthohantavirus patients, it should be considered whether the significantly lower AUC values of ADP, COL and TRAP in DENV cases are virus-specific or whether they can be attributed to viral (haemorrhagic fever) infections in general. Given the extent to which thrombopathy is a hallmark in DENV infections, it is likely to fit with only a small number of viral infections, orthohantavirus and DENV included. In any case, from our data it would seem that a clear clinical suspicion of DENV infection (i.e. by first applying WHO 2009 clinical decision rules) and low AUC ADP values on hospital admission relates to a prolonged hospital stay.

The exact route by which DENV induces thrombopathy, of which ADP, COL and TRAP values measured by MultiPlate are a representation needs further study. In recent studies, for instance, the modulation of DC-SIGN and FcYR2A receptor expression on thrombocytes, was suggested to have a protective effect because it prevents tissue damage due to thrombocyte aggregation [42]. While targets for ADP, TRAP and COL might still be active, our results could support such findings in a way that the ability to stimulate thrombocyte aggregation is still possible thru ADP, TRAP and COL, but the resulting cascade to aggregation is temporarily impaired Trends to a stable recovery of ADP, TRAP and COL induced platelet aggregation can be observed in the D1 data, as well as in the sparse data available from D7 +/− 48 h. Also, recent in-vitro studies have shown that there are morphological changes in thrombocytes during DENV infections with changes in angiogenic and inflammatory profiles. This could possibly support our findings of impaired coagulation when thrombocytes are externally stimulated by means of ADP, TRAP and COL. Future studies should investigate whether this for instance might be due to conformational changes in receptor binding sites [13].

Results reported in the MultiPlate are a reflection of the potency of thrombocytes to aggregate in a fixed amount of time. However, the results are also influenced by the absolute numbers of thrombocytes, as has been reported in previous studies [30, 43]. For this reason, the arbitrary thrombocyte count of at least 100 × 10^9^/L was chosen. Furthermore, the use of medication that could potentially interfere with MultiPlate reliability was considered as an exclusion criterion. Surprisingly, we found a positive and significant correlation for thrombocyte counts in the fever of another origin group versus ADP AUC at baseline. We could not replicate this correlation for the low ADP AUC values that seem to be specific to the DENV group, which strengthens our findings. Also, in this regard no significant observations could be made for the whole study group (data not shown). It might therefore be likely that the low ADP values that we observed are DENV-specific for those with a thrombocyte count of > 100 × 10^9^/L at baseline.

A significant proportion of the regions in which DENV is endemic comprises countries that are considered low- to middle-income and in which some of their healthcare systems are characterised by having limited resources, from both a diagnostic and financial point of view. In outbreak situations, in particular, when many people might be simultaneously in need of medical care, the number of patients presenting at a hospital can easily exceed its capacity. Fast, reliable and cost-effective diagnostics that, preferably, predict the need for admission and/or intensified follow-up, would thus be highly relevant in these settings. Once set-up and validated using blood of healthy controls, the MultiPlate offers a fast turn-around time of less than 10 min and at a cost that is comparable to routine haematology and biochemistry laboratory tests. The combination of decreased aggregation, leukopenia and clinical signs like headache for rapid DENV diagnosis should be further studied, but our results so far suggest a potential role of thrombocyte function tests. Its eventual complementary role to molecular or ELISA/rapid based DENV diagnostics would however need further study. For this, we suggest that future studies should include molecular techniques to detect acute DENV, be conducted in several continents and be complemented with molecular testing on other viral haemorrhagic fevers. This then does not only account for circulating DENV serotypes but also defines the validity of our results for other haemorrhagic fevers.

## Conclusion

In this explorative study of measuring whole blood aggregation of thrombocytes using ADP, COL and TRAP, we have found potentially relevant insights in thrombocyte function in acute DENV. While future exploration is needed, such tests potentially serve as an additional marker in acute DENV. These findings should be further explored in more extensive cohort studies or in outbreaks where the number of patients presenting to a hospital with symptoms of DENV exceeds the available hospital capacity.

### Supplementary information

**Additional file 1: Table S1.** Performance of NS1 rapid antigen test vs NS1 ELISA capture assay.

## Data Availability

Data is available upon reasonable request to the corresponding author. Supplementary data is available from Appendix A and Table S1: Performance of NS1 rapid antigen test vs NS1 ELISA capture assay.

## For all patients, the treating physician was requested to score the following dengue signs/symptoms (following the WHO dengue 2009 guidelines – see main text for reference)

- Headache.
- Leukopenia.
- Muscle/joint pain.
- Nausea/vomiting.
- Rash.
- Retro-orbital pain.
- Swollen glands.

Firstly, all subjects were tested for the presence of DENV NS antigen with a DENV NS1 rapid test (Panbio® Dengue Early Rapid) and DENV NS1 ELISA (Panbio® Dengue Early ELISA dengue NS1) according to the manufacturer’s specifications. Those that tested positive (i.e. above the manufacturer-specified and/or per calculated cut-off value according to the manufacturer’s instructions) were classified as “Dengue confirmed”.

Secondly, for other enrolled subjects, a differential diagnose list was made by the supervising infectious diseases specialist. Patients whose fever was probably attributable to a different cause, such as suspected pneumonia, urosepsis or typhoid fever, for example, were classified in the group “fever of another origin”.

Those admitted with an unclear cause of fever, due to having less pronounced symptoms, for example, were evaluated in accordance with dengue signs/symptoms as mentioned previously and, if accompanied by two or more positive signs/symptoms, were classified as “probable dengue”.

Patients in which DENV infection was more than likely, but who were negative for NS1 ELISA, were evaluated in accordance with dengue signs/symptoms and, if accompanied by two or more positive symptoms were classified as “probable dengue”.

## Abbreviations

DENV: Dengue virus
NS1: Non-structural protein 1
ELISA: Enzyme-linked immunosorbent assay
ADP: Adenosine diphosphate
TRAP-6: Thrombin receptor activator for peptide 6
COL: Collagen
AUC: Area Under the aggregation Curve
HC: Healthy controls
HFRS: Haemorrhagic Fever and Renal Syndrome
AU: Aggregation units
NSAIDs: Nonsteroidal anti-inflammatory drugs
