# Supplementary material for: Point-of-care thrombocyte function testing using multiple-electrode aggregometry in dengue patients: an explorative study
Source: BMC Infect Dis. 2020 Aug 6;20:580. doi: 10.1186/s12879-020-05248-4 (PMC7409667; doi:10.1186/s12879-020-05248-4)
Supplement: Supplementary file 1 — Additional file 1: Table S1. Performance of NS1 rapid antigen test vs NS1 ELISA capture assay. [file 12879_2020_5248_MOESM1_ESM.docx]

**Suppl. Table 1. Performance of NS1 rapid antigen test vs NS1 ELISA capture assay**

| **Patient** | **NS1 rapid** | **NS1 ELISA  index** | **NS1 ELISA  assay result** | **Match** | **Study group** |
| --- | --- | --- | --- | --- | --- |
| MUPL-001-RURI-F | neg | 0.664554 | neg | Yes | probable |
| MUPL-002-SHMA-F | neg | 0.094598 | neg | Yes | another origin |
| MUPL-003-SAAR-M | neg | -0.28143 | neg | Yes | probable |
| MUPL-005-YURY-F | neg | 0.113518 | neg | Yes | another origin |
| MUPL-006-JAIO-M | neg | 0.125343 | neg | Yes | another origin |
| MUPL-007-DIWI-F | neg | 0.089869 | neg | Yes | probable |
| MUPL-008-BUSO-M | neg | 0.096963 | neg | Yes | probable |
| MUPL-009-SOTO-M | neg | 0.087504 | neg | Yes | another origin |
| MUPL-010-BATI-F | neg | 0.082774 | neg | Yes | another origin |
| MUPL-011-DENI-F | neg | 0.089869 | neg | Yes | another origin |
| MUPL-012-DWTA-F | neg | -0.20102 | neg | Yes | another origin |
| MUPL-013-IHAM-M | neg | 0.127708 | neg | Yes | another origin |
| MUPL-014-ARNO-F | neg | 0.115883 | neg | Yes | another origin |
| MUPL-015-SUTO-M | neg | 0.111153 | neg | Yes | another origin |
| MUPL-016-WANG-F | neg | 0.092233 | neg | Yes | another origin |
| MUPL-017-ARTA-F | pos | 3.105193 | pos | Yes | confirmed |
| MUPL-018-MOAE-M | pos | 5.831993 | pos | Yes | confirmed |
| MUPL-019-NENI-F | neg | 0.118248 | neg | Yes | another origin |
| MUPL-020-ABNI-M | neg | -0.10642 | neg | Yes | another origin |
| MUPL-021-INNA-F | neg | 0.111153 | neg | Yes | another origin |
| MUPL-022-DATO-M | neg | 0.125343 | neg | Yes | probable |
| MUPL-023-NAHA-F | pos | 6.749598 | pos | Yes | confirmed |
| MUPL-024-CAWO-M | pos | 1.648378 | pos | Yes | confirmed |
| MUPL-025-ADWI-F | pos | 5.550563 | pos | Yes | confirmed |
| MUPL-026-SHNO-M | neg | 0.593605 | neg | Yes | another origin |
| MUPL-027-ANKO-M | neg | 0.186832 | neg | Yes | probable |
| MUPL-028-YUAN-F | neg | -0.20812 | neg | Yes | probable |
| MUPL-029-THNA-F | neg | 0.122978 | neg | Yes | another origin |
| MUPL-030-SUDO-F | neg | 0.196292 | neg | Yes | another origin |
| MUPL-031-SAAH-F | neg | 0.148993 | neg | Yes | probable |
| MUPL-032-YUAN-M | neg | 0.115883 | neg | Yes | probable |
| MUPL-033-RATA-M | pos | 5.661716 | pos | Yes | confirmed |
| MUPL-034-NUTI-F | neg | 0.101693 | neg | Yes | probable |
| MUPL-035-FITI-F | pos | 7.229685 | pos | Yes | confirmed |
| MUPL-036-DEKI-F | neg | -0.17028 | neg | Yes | probable |
| MUPL-037-HETA-F | pos | 3.015325 | pos | Yes | confirmed |
| MUPL-038-SAUS-M | neg | 0.134803 | neg | Yes | another origin |
| MUPL-039-PUNI-F* | neg | 0.127708 | neg | Yes | excluded* |
| MUPL-040-IMAT-M | neg | 0.125343 | neg | Yes | probable |
| MUPL-041-DINO-M | neg | 0.108788 | neg | Yes | probable |
| MUPL-042-AJTI-F | neg | 0.099328 | neg | Yes | probable |
| MUPL-043-MAYA-F | pos | 7.385772 | pos | Yes | confirmed |
| MUPL-044-DWNO-M | neg | -0.2294 | neg | Yes | probable |
| MUPL-045-INNA-F | neg | 0.099328 | neg | Yes | probable |
| MUPL-046-SENI-F | neg | 0.151357 | neg | Yes | probable |
| MUPL-047-SUYO-M | neg | 0.146628 | neg | Yes | another origin |
| MUPL-048-AIRI-F | neg | 1.326743 | pos | No | confirmed |
| MUPL-049-ISTO-M | neg | 0.130073 | neg | Yes | probable |
| MUPL-050-MIAP-M | neg | 0.118248 | neg | Yes | another origin |
| MUPL-051-MUKI-M | neg | 0.416233 | neg | Yes | probable |
| MUPL-052-GIVO-M | neg | -0.13244 | neg | Yes | probable |
| MUPL-053-ABNA-M | neg | 0.087504 | neg | Yes | probable |
| MUPL-054-ADNO-M | neg | 0.130073 | neg | Yes | another origin |
| MUPL-055-YEPH-M | neg | 0.137168 | neg | Yes | probable |
| MUPL-056-RORA-F | neg | 0.130073 | neg | Yes | another origin |
| MUPL-057-NADI-M | neg | 0.118248 | neg | Yes | probable |
| MUPL-058-SUNO-M | neg | 0.113518 | neg | Yes | another origin |
| MUPL-059-WAKA-M | neg | 0.156087 | neg | Yes | probable |
| MUPL-060-ASTO-M | neg | -0.10169 | neg | Yes | probable |
| MUPL-061-NUTI-F | neg | 0.137168 | neg | Yes | another origin |

* The subject was analysed for both NS1 tests, but excluded from the final analysis due to incomplete data.
